# Association between C-reactive protein-to-lymphocyte ratio and depression: A cross-sectional study

**DOI:** 10.1097/MD.0000000000047563

**Published:** 2026-02-06

**Authors:** Yanan Zhu, Ting Ao, Fengjie Li

**Affiliations:** a Emergency Department, Beijing Luhe Hospital, Capital Medical University, Beijing, PR China; b Department of Infectious Diseases, Beijing Luhe Hospital, Capital Medical University, Beijing, PR China.

**Keywords:** C-reactive protein-to-lymphocyte ratio, depression, inflammation, National Health and Nutrition Examination Survey

## Abstract

The inflammatory response contributes to the progression and prognosis of depression. The C-reactive protein-to-lymphocyte ratio (CLR) is increasingly recognized as a promising indicator of systemic inflammatory activity. Yet the relationship between CLR and depression is still uncertain. The conducted study aimed to investigate the potential link between CLR and depression. We analyzed 12,578 participants aged ≥20 years from National Health and Nutrition Examination Survey 1999 to 2010. CLR ([C-reactive protein mg/L]/[lymphocyte × 10^3^/µL]) was calculated from fasting blood. Depression was defined as Patient Health Questionnaire-9 ≥ 10. Five sequential logistic models adjusted for demographics, lifestyle and comorbidities; quartile and trend analyses were performed, followed by stratified assessments. Overall depression prevalence was 8.53%. Each 0.1-unit CLR increment was associated with 11% higher odds of depression in the fully adjusted model (odds ratio = 1.11; 95% confidence intervals 1.01–1.22). Participants in the highest CLR quartile showed 36% increased risk versus the lowest quartile (odds ratio = 1.36; 95% confidence intervals 1.10–1.66; *P*-trend = .004). Findings were consistent across sex, age, education, smoking and metabolic subgroups. Higher CLR, reflecting combined innate activation and adaptive suppression, is significantly and dose-dependently associated with depression. This inexpensive, routinely available biomarker may aid early risk detection and guide anti-inflammatory treatment strategies; prospective studies are needed to confirm causality.

## 1. Introduction

Depression is known as a broad spectrum of mental-health conditions marked by the loss of positive affect – evident as reduced interest and enjoyment in daily activities – an enduring low mood accompanied by a range of linked cognitive, emotional, behavioral, and physical signs.^[1]^ It becomes a prevalent mental health disorder that affects a significant portion of the population, especially in today’s increasingly stressful society, leading to substantial morbidity and mortality. Mental disorders impose a staggering global burden, with the World Health Organization’s 2023 report revealing that depression affects over 280 million people worldwide and is counted among the foremost causes of disability-adjusted life years, which may cost 12 billion productive workdays’ lost every year and nearly $147 billion because of depression and anxiety.^[2-4]^ The rapid expansion of online information exposure may exacerbate depressive symptoms by increasing social comparison, misinformation, and emotional contagion, thereby further complicating the public health burden of depression.

The persistent challenge in elucidating depression’s etiology has fueled interest in the neuroinflammatory hypothesis. Pioneering studies demonstrate that peripheral inflammatory mediators (e.g., interleukin [IL]-6, C-reactive protein [CRP]) disrupt blood–brain barrier integrity and influence nearly every pathophysiologic process implicated in depression, including neurotransmitter turnover, neuroendocrine regulation, and synaptic plasticity, thereby alter functional connectivity in mood-regulating neural circuits – a mechanistic link established by Miller et al in Nature Reviews Immunology^[5]^ and expanded by Kiecolt-Glaser et al in Nature Reviews Neuroscience.^[6]^ Following studies have pointed to a possible connection between inflammation and depression, with raised levels of inflammation-related biomarkers, exemplified by CRP being observed in individuals with depressive symptoms.^[7]^ Furthermore, resolution of depression-like behaviors is an active, T-cell-dependent process that hinges on meningeal IL-10 induction, and a defect in this pathway may hinder recovery and promote major depression disorder.^[8]^

As a well-established inflammatory biomarker, CRP is indispensable for evaluating infections and diverse inflammatory conditions,^[9]^ whereas lymphocytes are key constituents in immunity and rapidly change in number whenever the balance shifts toward activation or suppression.^[10]^ Building on this insight, composite hematologic indices such as the neutrophil-to-lymphocyte ratio (NLR) and platelet-to-lymphocyte ratio (PLR) have proven to be cost-effective inflammatory markers in oncology and cardiology, yet their application in psychiatry remains limited. Recent years, elevated NLR,^[11,12]^ PLR,^[13,14]^ and monocyte-to-lymphocyte ratio^[15]^ have been directly employed to associate with depression. Around 2020, a more stable innovative indicator – the CRP-to-lymphocyte ratio (CLR) – was introduced to capture the interplay between systemic inflammation and immune responsiveness; its feasibility was first demonstrated in pancreatic cancer prognosis.^[16]^ Subsequent studies have linked higher CLR values to worse clinical outcomes in myocardial infarction,^[17]^ severe acute respiratory syndrome coronavirus 2 pneumonia,^[18]^ periprosthetic joint infection,^[19]^ and several malignancies, underscoring CLR’s broad utility as a prognostic and diagnostic biomarker. Given that inflammatory processes and immune dysregulation lie at the heart of depression pathophysiology, CLR could theoretically reflect vulnerability to the disorder. Despite the biological plausibility, large-scale epidemiological evidence directly connecting CLR with depression risk is still lacking. We therefore conducted a population-based cohort study to determine whether elevated CLR independently is associated with incident depression, even after adjustment for traditional confounders – age, sex, lifestyle variables, comorbidities, and so on.

## 2. Materials and methods

### 2.1. Study population

This study was approved by the Ethics Committee of Beijing Luhe Hospital, Capital Medical University. The National Health and Nutrition Examination Survey (NHANES) program is run by the National Center for Health Statistics (NCHS) – a division of the Centers for Disease Control and Prevention – to collect data about the health of all ages and serves as one of the most comprehensive and reliable studies in the United States to evaluate the general health and dietary conditions of the country’s residents. The survey employs a multistep, intricate, probability-based sampling strategy to select a representative sample of the noninstitutionalized US population. The data are released biennially and are publicly available, and the data collection is conducted in 2 phases: a household interview on demographics, health habits, and health care access information and a mobile examination center (MEC) visit on a comprehensive physical examination, laboratory tests, and dietary interviews. Therefore, the survey measures a wide range of health indicators, including a series of objective biomarkers, nutritional status and health conditions, such as obesity, diabetes, heart disease, which have been instrumental in identifying emerging health issues, tracking disease trends, evaluating the effectiveness of public health interventions, and monitoring and driving changes in how public policy supports good health. The Research Ethics Review Board of NCHS granted approval for all survey protocols, and all participants provided informed consent. Comprehensive details about the survey methodologies and protocols can be found on the official NCHS website.

Our study follows and meets the exemption criteria in Article 32 of the Ethical Review Methods for Life Science and Medical Research Involving Human Beings: we used public data obtained legally or observed public behavior without interfering with it and all data were anonymized for research. We legally obtained and utilized data from public databases and made sure that our research didn’t affect public behavior and that all data were kept and analyzed anonymous, meeting the ethical requirements. A cross-sectional design were adopted in this research with NHANES’ data between 1999 and 2010 which only including adult participants. Meanwhile, we excluded 1299 pregnant individuals and all individuals with incomplete information, including 3884 with missing CRP records, 130 with missing lymphocyte data, 13,924 with missing depression information, and 649 with other missing covariates information.

### 2.2. C-reactive protein-to-lymphocyte ratio

In this study, the exposure variable CLR was calculated as CRP (mg/L) divided by lymphocyte count (1000 cells/µL).^[20]^ Whole blood specimens collected at the NHANES MEC was processed under strict quality-control protocols before CLR was computed.

### 2.3. Depression

In the NHANES database, depressive status was assessed with the 9-item Patient Health Questionnaire (PHQ-9).^[21]^ This standardized screening tool is completed by participants at the MEC via a computer-assisted personal interview system. The PHQ-9 records the frequency of depressive symptoms experienced during the past 2 weeks and each item is scored from 0 (not at all) to 3 (nearly every day), generating an aggregate range of 0 to 27. Depression was defined as a PHQ-9 total score ≥ 10, indicating clinically significant depressive symptoms.

### 2.4. Covariates

All date collection and documentation were performed by highly trained NHANES team and medical professionals. The study captured 3 broad data domains: demographic and lifestyle variables: age (years), sex, race/ethnicity, marital status, education, poverty–income ratio (PIR), alcohol use, and smoking status; comorbidities: hyperlipidemia, hypertension, and diabetes; and laboratory indices: counts of segmented neutrophils, eosinophils, monocyte, basophils, and platelets (all in 10^3^ cells/µL), plus red blood cell count (10^6^ cells/µL).

Gender/Sex was coded as male or female. Ethnicity/Race was partitioned into 5 categories: non-Hispanic White, non-Hispanic Black, other Hispanic, Mexican American, or other races. Three levels – above high school, high school/general educational development equivalent and below high school – was used to describe educational attainment. Marital status was dichotomized as married/cohabiting or living alone. Alcohol use and smoking status were labeled current, former, or never. PIR was split into ≤1.30, 1.31 to 3.50, and>3.50.

Hyperlipidemia was defined as total cholesterol ≥ 5.2 mmol/L, triglycerides ≥ 1.7 mmol/L, low-density lipoprotein cholesterol ≥ 3.4 mmol/L, or high-density lipoprotein cholesterol < 1.0 mmol/L. Hypertension was diagnosed if the average systolic blood pressure ≥ 140 mm Hg or the average diastolic blood pressure ≥ 90 mm Hg. Or it was identified as a prior clinical diagnosis of hypertension or any use of antihypertensive drugs. Diabetes was deemed present when any one of the following applied: fasting glucose ≥ 7.0 mmol/L, random blood glucose ≥ 11.1 mmol/L, 2-hour post-load glucose ≥ 11.1 mmol/L on an oral glucose tolerance test, glycated hemoglobin (HbA1c) ≥ 6.5%, current insulin or other glucose-lowering therapy, or a diagnosed diabetes recorded by a physician.

### 2.5. Statistical analysis

Drawing on prior literature and clinical practice, we obtained a series of demographic and laboratory variables for all participants. No covariate had >10% missing values thanks to the ample sample size. Given this low level of missing data, we directly elected to exclude records with absent values. Categorical variables in the depression and non-depression groups were reported as percentages (%). Depending on their distributional characteristics, continuous variables are presented either as means ± standard deviations when approximately normal, or as medians together with their interquartile ranges when the data were skewed. Differences across groups were evaluated with the 1-way analysis of variance (for normally distributed continuous outcomes), the Kruskal–Wallis test (for skewed continuous outcomes), or the chi square test (for categorical variables), whichever was most appropriate to the data type and distribution. In the follow-up analyses exploring the association with depression, CLR was approached as a continuous variable (per 0.1-unit increment) or as a categorical variable based on quartiles. Multivariable logistic regression models were used to estimate odds ratios (ORs) and their corresponding 95% confidence intervals. Five sequential models were constructed: model 1 was unadjusted; model 2 further adjusted for age, sex, and race/ethnicity; model 3 additionally included education level, marital status, alcohol consumption, smoking status, and PIR; model 4 added comorbidities (hyperlipidemia, hypertension, and diabetes) to model 3; and model 5 represented the fully adjusted specification.

Stratified analyses were additionally performed according to sex, age (20–59 years vs ≥60 years), education level, smoking status (nonsmokers vs smokers), alcohol consumption (nondrinkers vs drinkers), hyperlipidemia, hypertension, and diabetes to assess the consistency of the association between CLR and depression across subgroups.

All analyses were conducted with Free Statistics software version 2.1 and R version 4.2.2 (R Foundation for Statistical Computing, http://www.R-project.org). Statistical significance was set at a 2-sided *P*-value < .05.

## 3. Results

### 3.1. Baseline characteristics

**Figure 1.**
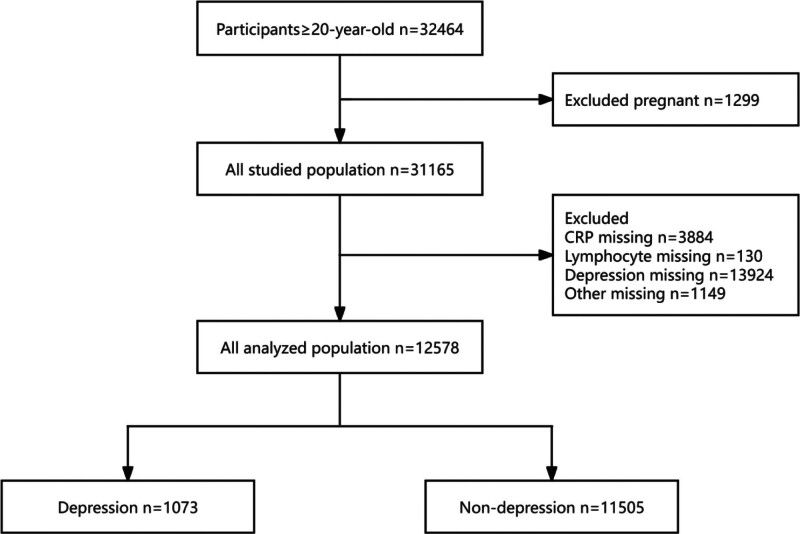
Flow chart of participants selection. CRP = C-reactive protein.

The total number of individuals successfully enrolled in the study is 12,578 from 1999 to 2010 (Fig. 1), including 49.3% male and 50.7% female. The mean age was 49.9 years, with a standard deviation of 17.8 years. Of these, the overall prevalence of depression was 8.53%. Of note is that participants in the depression group tended to be younger (mean age 47.7 years vs 50.1 years, *P* < .001), predominantly female (63.6% vs 48%, *P* < .001), and more likely to be unmarried or in other marital statuses (51.7% vs 37.3%, *P* < .001). They also have a lower income level as well as lower educational attainment and are more frequently current smokers and drinkers. Additionally, participants with depression exhibit a greater incidence of hyperlipidemia, hypertension, and diabetes. Moreover, individuals diagnosed with depression exhibited both reduced lymphocyte counts and lower CRP levels relative to their nondepressed counterparts (Table 1).

**Table 1:** Weighted baseline characteristics of participants in the NHANES 1999–2010 cycles.

Patient characteristic	Total (n = 12,578)	Non-depression (n = 11,505)	Depression (n = 1073)	*P*-value
Demographic information				
Age, Mean ± SD, yr	49.9 ± 17.8	50.1 ± 18.0	47.7 ± 15.4	<.001
Sex, n (%)				<.001
Male	6372 (50.7)	5981 (52)	391 (36.4)	
Female	6206 (49.3)	5524 (48)	682 (63.6)	
Race/ethnicity, n (%)				<.001
Non-Hispanic White	6445 (51.2)	5950 (51.7)	495 (46.1)	
Non-Hispanic Black	2409 (19.2)	2178 (18.9)	231 (21.5)	
Mexican American	2235 (17.8)	2041 (17.7)	194 (18.1)	
Other Hispanic	994 (7.9)	881 (7.7)	113 (10.5)	
Other	495 (3.9)	455 (4)	40 (3.7)	
Marital status, n (%)				<.001
Married/Living with a partner	7728 (61.4)	7210 (62.7)	518 (48.3)	
Never married/Other	4850 (38.6)	4295 (37.3)	555 (51.7)	
Poverty income ratio, n (%)				<.001
≤1.3	3699 (29.4)	3140 (27.3)	559 (52.1)	
1.3–3.5	4848 (38.5)	4492 (39)	356 (33.2)	
>3.5	4031 (32.0)	3873 (33.7)	158 (14.7)	
Educational level, n (%)				<.001
Less than high school	6449 (51.3)	5765 (50.1)	684 (63.7)	
High school or equivalent	3521 (28.0)	3229 (28.1)	292 (27.2)	
Above high school	2608 (20.7)	2511 (21.8)	97 (9)	
Smoke, n (%)				<.001
Never	6505 (51.7)	6085 (52.9)	420 (39.1)	
Former	3245 (25.8)	3024 (26.3)	221 (20.6)	
Current	2828 (22.5)	2396 (20.8)	432 (40.3)	
Drink, n (%)				<.001
Never	1622 (12.9)	1489 (12.9)	133 (12.4)	
Former	2499 (19.9)	2233 (19.4)	266 (24.8)	
Current	8457 (67.2)	7783 (67.6)	674 (62.8)	
Disease status				
Hyperlipidemia, n (%)	9260 (73.6)	8429 (73.3)	831 (77.4)	0.03
Hypertension, n (%)	5284 (42.0)	4765 (41.4)	519 (48.4)	<.001
Diabetes, n (%)	2205 (17.5)	1938 (16.8)	267 (24.9)	<.001
Biochemical indicators				
Segmented neutrophils number, Mean ± SD, 1000 cells/µL	4.3 ± 1.9	4.2 ± 1.9	4.6 ± 1.9	<.001
Lymphocyte number, Mean ± SD, 1000 cells/µL	2.2 ± 1.3	2.2 ± 1.3	2.3 ± 0.9	.009
Monocyte number, Mean ± SD, 1000 cells/µL	0.6 ± 0.2	0.5 ± 0.2	0.6 ± 0.2	.365
Eosinophils number, Median (IQR), 1000 cells/µL	0.2 (0.1, 0.3)	0.2 (0.1, 0.3)	0.2 (0.1, 0.3)	.241
Basophils number, Median (IQR), 1000 cells/µL	0.0 (0.0, 0.1)	0.0 (0.0, 0.1)	0.0 (0.0, 0.1)	.004
Red blood cell count, Mean ± SD, million cells/µL	4.7 ± 0.5	4.7 ± 0.5	4.6 ± 0.5	<.001
Platelet count, Mean ± SD, 1000 cells/µL	260.2 ± 70.0	259.2 ± 69.5	270.9 ± 74.5	<.001
C-reactive protein,Median (IQR), mg/L	2.0 (0.8, 4.7)	1.9 (0.8, 4.5)	2.8 (1.0, 6.3)	<.001

IQR = interquartile range, NHANES = National Health and Nutrition Examination Survey, SD = standard deviation.

### 3.2. Association between CLR and depression

Table 2 presents the relationship between CLR and depression across 5 distinct models, illustrating how this association remains consistent regardless of adjustments. In all models, CLR shows a significant positive association with depression. In model 1, a 0.1-unit increase in CLR levels was associated with an OR of 1.18 (95% confidence intervals (CI), 1.08–1.29; *P* < .001) for depression. This association remained significant after adjusting for additional confounding factors in models 2 to 5, with the OR decreasing to 1.11 (95% CI, 1.01–1.22; *P* = .035) in model 5.

**Table 2:** Association between 0.1 CLR and depression.

	Model 1		Model 2		Model 3		Model 4		Model 5	
	OR (95%CI)	*P*-value	OR (95%CI)	*P*-value	OR (95%CI)	*P*-value	OR (95%CI)	*P*-value	OR (95%CI)	*P*-value
0.1 CLR	1.18 (1.08–1.29)	<.001	1.19 (1.09–1.31)	<.001	1.16 (1.06–1.27)	.001	1.14 (1.04–1.25)	.007	1.11 (1.01–1.22)	.035
0.1 CLR Quartiles										
Q1	1 (Ref)		1 (Ref)		1 (Ref)		1 (Ref)		1 (Ref)	
Q2	1.18 (0.97–1.43)	.105	1.25 (1.02–1.52)	.03	1.22 (1–1.5)	.052	1.18 (0.96–1.45)	.116	1.18 (0.96–1.45)	.112
Q3	1.5 (1.24–1.82)	<.001	1.56 (1.28–1.89)	<.001	1.44 (1.18–1.77)	<.001	1.35 (1.1–1.66)	.004	1.35 (1.1–1.66)	.004
Q4	1.75 (1.45–2.12)	<.001	1.76 (1.45–2.14)	<.001	1.56 (1.28–1.9)	<.001	1.39 (1.13–1.7)	.002	1.36 (1.1–1.66)	.004
Trend test		<.001		<.001		<.001		.001		.002

Model 1 was crude model; model 2 was adjusted for age, sex, race/ethnicity; model 3 was adjusted for model2 + marital status, poverty–income ratio, educational level, smoking status, drinking status; model 4 was adjusted for model3 + hyperlipidemia, hypertension, diabetes; model 5 was adjusted for model4 + segmented neutrophils number, platelet count, eosinophils number, monocyte number, basophils number, red blood cell count.

CI = confidence interval, CLR = C-reactive protein-to-lymphocyte ratio, IQR = interquartile range, NHANES = National Health and Nutrition Examination Survey, OR = odds ratio, Ref = reference, SD = standard deviation.

When CLR levels were analyzed as quartiles, participants in the fourth quartile (Q4) had the highest risk of depression, with an OR of 1.75 (95% CI, 1.45–2.12; *P* < .001) in model 1. This risk remained elevated in all subsequent models, with an OR of 1.36 (95% CI, 1.10–1.66; *P* = .004) in model 5. However, individuals in the second quartile (Q2) have a 18% to 25% higher odds of depression, which is not statistically significant in almost all models, compared to those in the first quartile (Q1). A significant trend test for CLR quartiles was observed in all models (*P* < .001 to *P* = .002).

### 3.3. Subgroup analyses

**Figure 2.**
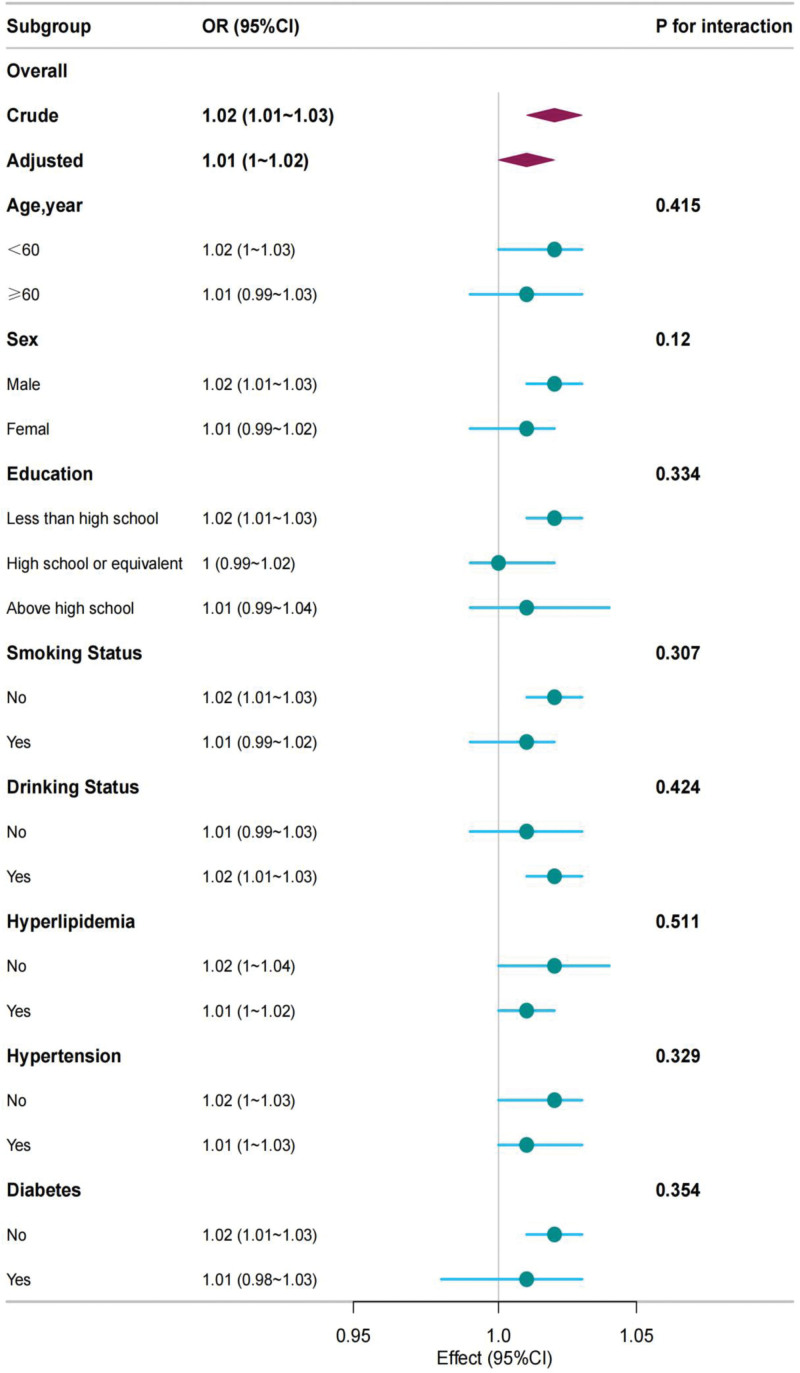
Subgroup analyses for the association of 0.1 C-reactive protein-to-lymphocyte ratio and depression. CI = confidence interval, OR = odds ratio.

To explore the heterogeneity of the association between CLR levels and depression, a series of stratified analyses were performed across various demographic and clinical subgroups. However, no statistically significant interaction effects were identified within any of these subgroups (Fig. 2).

## 4. Discussion

Using a nationally representative sample of US adults from the NHANES database and controlling for multiple potential covariates, we observed a positive association between CLR and depression; specifically, higher CLR values were associated with an increased risk of depression, with evidence of a dose–response relationship. Further stratified assessments corroborated the robustness of these findings.

CRP, as an acute-phase protein produced by hepatocytes, is a well-validated systemic inflammatory biomarker. Circulating CRP concentrations are markedly elevated during depressive episodes, reflecting a state of heightened systemic inflammation.^[22,23]^ Concomitantly, pro-inflammatory cytokines – including IL-6 and tumor necrosis factor-α – are released, synergistically disrupting blood–brain barrier integrity and triggering microglial activation.^[24]^ These processes collectively disturb neurotransmitter metabolism (such as a decrease in serotonin) and produce the neurotoxic metabolite, quinolinic acid, ultimately leading to neuronal and glial injury and the emergence of core depressive symptoms such as anhedonia and cognitive dysfunction (depressed mood and cognitive decline).^[25]^ Singh and colleagues further demonstrated that both first-episode and recurrent major depression are characterized by neutrophilia, elevated CRP, and eosinopenia, indicative of exaggerated innate immune system activation. Further more, immune imbalance is evident in the form of lymphopenia, which reflects suppression of adaptive immunity. Increased lymphocyte apoptosis, their migration into inflamed tissues, myelosuppressive, and hormone-mediated immunosuppression all contribute to this decline in lymphocyte counts.^[26,27]^ Multiple meta-analyses have shown that several lymphocyte-based inflammatory indices – such as NLR, PLR, and monocyte-to-lymphocyte ratio – are associated with both the onset and progression of depression as well as with suicidal ideation in depressed patients.^[28–30]^ Maya Amitai’s team reported that, in a sample of 91 youths (mean age 13.9 ± 2.4 years), depressed participants with a documented suicide attempt presented significantly higher baseline NLR and PLR than those without such history. After adjustment for sex, depression severity, and IL-6 concentrations, the elevation in NLR still emerged as an independent correlate of prior attempt (β = 1.247, *P* = .019; OR [95% CI] = 3.478 [1.230–9.841]).^[31]^ Based on these findings, CLR shows promise as an innovative biomarker capable of tracking the progression of depressive disorders.

The results of this study demonstrate that elevated CLR is significantly associated with increased risk of depression, and this association persists after adjusting for multiple potential confounders. CLR correlates with systemic inflammatory responses and immune status, helping to clarify the mechanisms underlying its link to depression risk. While CRP alone is susceptible to transient infections and lymphocyte counts alone are influenced by circadian rhythms, their ratio provides a more stable reflection of chronic inflammation. As an integrated index of inflammation and immune balance, CLR captures both innate immune activation (elevated CRP) and adaptive immune suppression (reduced lymphocytes), making it more sensitive than either marker alone in detecting immune dysregulation associated with depression. Such dysregulation activates the hypothalamic-pituitary-adrenal axis and reduces glucocorticoid receptor sensitivity, amplifying inflammatory signaling and playing a pivotal role in the pathogenesis of depression.^[32]^ Building on the findings of Singh et al published in the Journal of Neuroinflammation, increased CLR reflects an imbalance between peripheral inflammatory activation and immune suppression, offering new insight into the pathophysiology of depression. Notably, Singh’s study observed higher lymphocyte counts in patients with recurrent depression, suggesting compensatory immune activation during chronic stages and supporting the notion that CLR dynamics are linked to the phase of the disorder.^[23]^ Collectively, CLR – quantifying peripheral inflammation-immune imbalance – participates in and amplifies depressive pathology by disrupting the blood–brain barrier, eliciting central inflammation, and perturbing neurotransmitter metabolism. It therefore holds promise as a novel biomarker for early risk detection and treatment monitoring in depression and may guide immunologically targeted therapies.

The strengths of this study include providing new evidence linking CLR to depression risk. The research population is well-defined and relatively homogeneous, and it is derived from a large, nationally representative sample of adult Americans. This method can effectively adjust for confounding factors and minimize the impact of these factors on the results to the greatest extent. And routine blood tests are inexpensive and widely available, making them feasible in virtually any clinical setting. Leveraging these biomarkers could enable earlier, personalized interventions, optimizing resource allocation, and ultimately reducing disease burden. Our findings could equip clinicians with evidence to craft individualized surveillance and therapeutic plans that better match each patient’s needs.

Several limitations, however, merit consideration. First, the retrospective nature of the study inherently limits both the capacity to eliminate confounding influences and the possibility of establishing causal relationships. Second, because of the inherent structure of the NHANES database, depression status was ascertained primarily through self-reported screening instruments, which may introduce reporting bias. Third, CRP and lymphocyte levels are influenced by multiple factors – including current immune status as well as any recent episodes of acute infections – that could not be completely accounted for in the present analyses. Moreover, we lacked information on medication use, history of psychotherapy, or central inflammatory markers; although regression modeling, subgroup analyses, and sensitivity checks were employed, residual or unmeasured confounding cannot be ruled out. Longitudinal studies are therefore warranted to establish causal relationships and to elucidate the underlying mechanisms linking CLR to depression.

Several potential sources of bias should be considered when interpreting our findings. First, due to the cross-sectional design, reverse causation cannot be excluded; depressive status itself may influence inflammatory markers through behavioral changes, neuroendocrine dysregulation, or medication use. Second, depression was assessed using the PHQ-9 questionnaire rather than clinical diagnostic interviews, which may introduce misclassification or reporting bias. Third, CRP and lymphocyte levels are subject to short-term fluctuations related to acute infections, stress, or circadian variation, which may not be fully captured despite adjustment for multiple covariates. In addition, residual confounding from unmeasured factors – such as antidepressant use, psychotherapy, dietary patterns, or central nervous system inflammation – remains possible. Finally, although NHANES employs a nationally representative sampling strategy, selection bias may arise from missing laboratory or questionnaire data. These limitations underscore the need for longitudinal studies to clarify temporal relationships and causal mechanisms.

## 5. Conclusions

This study highlights the significant association between elevated CLR levels and depression. The results suggest that inflammation may be a contributing factor in the pathogenesis of depression and CLR may be a useful biomarker for depression. Subsequent researches should prioritize elucidating the mechanistic pathways and developing anti-inflammatory therapeutic strategies for the treatment of depressive disorders. Additionally, further studies are needed to validate these findings in different populations and settings.

## Acknowledgments

We thank Dr Jie Liu (Department of Vascular and Endovascular Surgery, Chinese PLA General Hospital) and his team for statistical support, study-design advice, and manuscript comments, and the NHANES staff for the open data.

## Author contributions

**Conceptualization:** Yanan Zhu, Ting Ao, Fengjie Li.

**Data curation:** Yanan Zhu, Ting Ao, Fengjie Li.

**Formal analysis:** Yanan Zhu, Ting Ao, Fengjie Li.

**Funding acquisition:** Fengjie Li.

**Investigation:** Fengjie Li.

**Writing – original draft:** Yanan Zhu, Fengjie Li.

**Writing – review & editing:** Yanan Zhu, Fengjie Li.
